# Application and Possible Mechanism of Microbial Fermentation and Enzyme Catalysis in Regulation of Food Flavour

**DOI:** 10.3390/foods14111909

**Published:** 2025-05-28

**Authors:** Feng Wang, Mingtong Wang, Ling Xu, Jingya Qian, Baoguo Xu, Xianli Gao, Zhongyang Ding, Kai Cui

**Affiliations:** 1 School of Food and Biological Engineering, Jiangsu University, Zhenjiang 212013, China; 2222418070@stmail.ujs.edu.cn (M.W.); lxu@ujs.edu.cn (L.X.); qianjingya@ujs.edu.cn (J.Q.); xbg@ujs.edu.cn (B.X.); 2 Key Laboratory of Carbohydrate Chemistry and Biotechnology, Ministry of Education, School of Biotechnology, Jiangnan University, Wuxi 214122, China; bioding@163.com; 3 Institute of Feed Research, Chinese Academy of Agricultural Sciences, Beijing 100081, China; cuikai@caas.cn

**Keywords:** food flavor, microorganisms, enzymes, fermentation, mechanism

## Abstract

Flavor compounds are key determinants of food sensory quality, originating from natural sources, processing, or artificial additives. Although physical and chemical methods can effectively enhance food flavor, microbial fermentation and enzyme catalysis technology possess good potential in food flavor regulation due to their mild reaction conditions and high safety. In addition, the high efficiency and specificity of enzymes help to shorten the production cycle and accurately regulate food flavor. This review focuses on the application and regulation mechanism of bacteria, yeast, other fungi, and mixed microbe fermentation systems in flavor production. The utilization and catalytic reaction schemes of oxidoreductases, transferases, and hydrolases in flavor regulation are also deeply explored, and suggestions for the application of microbial fermentation and enzyme catalysis technology in flavor regulation are discussed.

## 1. Introduction

Food flavor substances refer to compounds that can impart specific flavor to food. These compounds can stimulate human olfactory and taste organs to produce sensory reactions such as aroma and taste. Some raw materials themselves naturally have flavor substances [1]. For example, fruits contain a variety of volatile substances such as esters and alcohols that give them a sweet smell, while amino acids, peptides, and nucleotides in meat are important flavoring substances of meat flavor. Food also produces flavor substances during storage and processing. Fermentation is an important process used to produce flavor substances [2,3]. In the process of brewing, microorganisms such as yeast convert sugars into alcohol and produce various flavor substances such as esters and higher alcohols. In addition, the flavor of food can also be enhanced to a certain extent by artificially adding edible spices and seasonings.

Flavor substances are key factors in determining the flavor of food, and good flavor can improve the overall sensory quality of food [4]. However, some raw materials have unpleasant flavors, and adding appropriate flavor substances can improve the taste. At present, the development of plant-based meat imitations using soybean protein is hindered by the beany smell produced during extrusion processing [5]. Studies have shown that malt and flavonoids can reduce the beany flavor of the product and significantly improve its sensory quality [5,6,7]. Studies have reported that flavor substances have successfully simulated the taste of mango and Hami melon [8,9].

Some researchers have found that physical methods such as ultrasound and embedding can effectively improve the flavor of meat products, plant-based artificial meat, and other foods [10,11,12,13,14,15]. It is also a good choice to directly synthesize specific flavor substances such as esters and aldehydes through chemical reactions or directly add spices and flavor enhancers to food. Rocha et al. evaluated the ability of monosodium glutamate (MSG), disodium inosinate (IMP), disodium guanylate (GMP), and monoammonium glutamate (MAG) to enhance saltiness in aqueous solutions containing different concentrations of sodium chloride. Studies have shown that MAG and MSG have a greater ability to enhance saltiness than IMP and GMP at any sodium chloride concentration [16]. Although these methods can quickly improve food flavor, the excessive consumption of flavor enhancers is not conducive to human health, and there is also a risk of chemically synthesized flavor substances reacting with other components to produce harmful substances. The use of microorganisms or enzymes can address this problem. Microbial fermentation is a green and environmentally friendly food processing method. It uses the various metabolic activities of microorganisms to decompose macromolecules such as proteins, carbohydrates, and fats in food to obtain different metabolites and produce unique flavors [17,18]. This method does not require the addition of artificially synthesized flavor substances, and the reaction conditions are mild, usually carried out at room temperature and pressure, which meets the requirements of green production and reduces environmental pollution. As biocatalysts, enzymes are safe and the enzymatic reaction is usually carried out under mild conditions, which helps to retain the original nutrients of food. The high efficiency and specificity of enzymes are also conducive to shortening the production cycle while avoiding unnecessary by-products and accurately regulating food flavor.

In recent years, the application of microbial fermentation and enzyme catalysis technology in food flavor improvement has attracted considerable attention and has been systematically discussed in many reviews. Liu et al. reviewed the existence of probiotic *Bacillus* strains in the microbial composition of liquid, semi-solid, and solid-state fermented food, and statistically analyzed the single fermentation and mixed fermentation of the probiotic *Bacillus* as a food starter. The researchers found that the use of probiotic *Bacillus* strains in food fermentation enhanced the flavor and texture of the product, increased nutritional value, and improved safety. In addition, these strains contribute to the metabolism of food raw materials, shorten the fermentation time, and provide a new way to reduce the salt content in fermented foods [19]. Wang et al. reviewed the evolution of microbial community structure during food fermentation, as well as the key volatile compounds affecting food flavor and their potential relationships. They found that to improve the quality of traditional fermented foods, it is necessary to break the metabolic regulation mechanism of microorganisms and eliminate the accumulation of by-products to achieve the efficient control of certain flavor products [2]. Ye et al. reviewed the mechanism of endogenous and exogenous lipases and lipoxygenases in fermented fish products and their effects on the main volatile flavor substances in various fermented fish products. The authors found that neutral and acidic lipases mainly catalyze the hydrolysis of triglycerides to produce free fatty acids (FFAs) and other intermediate metabolites. Lipoxygenase catalyzes the oxidation of polyunsaturated fatty acids produced during lipolysis. Lipase and lipoxygenase play a synergistic role in the fermentation process, producing a variety of volatile flavor components such as alcohols, aldehydes, esters, ketones, and acids, thereby enriching the flavor of fermented fish products [20].

Most existing studies focus on the role of single microorganisms or specific enzymes, and the synergistic mechanism of different microbial communities and enzymes in food flavor regulation has not been systematically explained. Therefore, this study focused on the application and mechanism of bacteria, yeasts, other fungi, and mixed bacteria fermentation systems in flavor regulation, as well as the application and catalytic reaction schemes of oxidoreductases, transferases, and hydrolases in flavor regulation, to provide a systematic theoretical basis for the application of microbial fermentation and enzyme catalysis technology in flavor regulation.

## 2. Application of Microbial Fermentation in Regulation of Food Flavor

### 2.1. Applications of Different Microorganisms

#### 2.1.1. Bacteria

Lactic acid bacteria (LAB) and *Bacillus* are commonly used fermentation microorganisms in the field of food flavor regulation [21,22].

LAB are a class of Gram-positive bacteria that can ferment sugars to produce lactic acid [23]. Most have no spores and are important probiotics [24]. They not only exhibit antibacterial and antioxidant activity, but also promote the decomposition of proteins and lipids, and produce flavor precursors such as free amino acids or FFAs, thus significantly affecting the flavor characteristics of food [2]. LAB improves the flavor of yogurt by producing volatile flavor substances such as lactic acid and acetaldehyde [25]. In cheese fermentation, LAB mainly decompose proteins and fats to generate free amino acids and fatty acids to form complex flavors [26]. LAB fermentation is a widely used biotechnology process. This fermentation method increases the nutritional value and flavor of food, reduces anti-nutritional compounds, promotes antioxidant and immune responses, and prolongs the shelf life of products [27,28]. *Lactobacillus plantarum* is one of the most commonly used LAB to improve food flavor [29,30]. Studies have shown that *L. plantarum* fermentation can increase the content of bioactive components such as organic acids and phenols and volatile compounds such as acids, alcohols, and ketones in lily beverages, carrot pulp, and asparagus juice; enhance the flavor of fruit juice beverages; reduce the content of terpenes, furans, and other undesirable flavor substances; and improve the overall acceptability of products [27,31,32]. Kombucha is a traditional beverage produced through the fermentation of sweet black tea by bacteria and symbiotic yeast [33,34,35]. The introduction of *L. plantarum* reduces the total acid and acetic acid content, promotes the production of alcohols and esters, and enriches the fruity and floral characteristics of kombucha [33]. *L. plantarum* was used as an enhanced starter in the post-fermentation stage of surimi products, and the ester content increased by 20%, especially ethyl caprylate, which further enhanced the flavor of surimi products. In addition, the content of biogenic amines and nitrite decreased by 17% and 37%, respectively, during fermentation [36]. Xu et al. and Zheng et al. found that the inoculation of *L. plantarum* can significantly shorten the fermentation cycle of radish pickles, reduce the content of nitrite and conditional pathogenic bacteria, and improve the edible safety of pickles [37,38]. In addition, the content of sweet amino acids (threonine and glycine) in pickles fermented via inoculation was low, and the content of some bitter amino acids (histidine and arginine) and volatile substances was high, which may enhance the palatability of pickles [37,39]. Zhang et al. found that the artificial inoculation of *Leuconostoc mesenteroides* and *Lactobacillus paracasei* rapidly fermented the production of northeast sauerkraut and promoted the catabolism and synthesis of amino acids, thereby improving the flavor and quality [40]. *L. plantarum* also has significant advantages in enhancing the umami flavor of mushrooms. Chen et al. found that the acidity, total free amino acid content, and total flavor nucleotides of the fermentation broth showed an upward trend throughout the fermentation process [41]. Chen et al. focused on the effect of *L. plantarum* fermentation on the umami flavor substances of *Lentinula edodes*. The results showed that except for succinic acid and fumaric acid, the content of all umami substances increased with the extension of fermentation time [42]. In addition, the researchers posited that the fermentation of *L. plantarum* GDM1.191 could improve the umami flavor of *Lentinula edodes* mainly because most of the free amino acids and 5′-nucleotides increased after fermentation had strong umami flavor characteristics [42].

Bean products generally have bitter, astringent, and beany odors [43]. LAB fermentation can reduce such odors and improve the flavor of beans. Studies have shown that *Lactobacillus johnsonii*, *Lactobacillus fermentum*, *L. plantarum*, *Corynebacterium glutamicum*, and other LAB can effectively improve the flavor of pea protein, mung bean, soybean protein isolate (SPI), and other legume foods [44,45,46,47,48,49]. During the fermentation process, these LAB increased the types and contents of bioactive substances such as amino acids and peptides, produced aroma components such as alcohols and ketones, and reduced beany flavor components such as aldehydes, thus effectively improving the overall flavor of bean products [44,45,46,47,48,49].

*Bacillus* is a Gram-positive spore genus that is widely found in the natural environment [2,50]. Most of the *Bacillus* species, such as *Bacillus licheniformis* and *Bacillus subtilis*, have shown probiotic properties [19,51,52]. Probiotic Bacillus have been shown to produce cellulase, amylase, protease, and lipase [2,53]. In addition, these strains can also produce antibacterial metabolites such as bacteriocins and peptides, thereby inhibiting the growth and reproduction of harmful bacteria [19,54]. It has been found that *probiotic Bacillus* strains can effectively solve the problems of poor flavor and long fermentation time of fermented products caused by the lack of certain functional genes and the insufficient metabolic capacity of *Lactobacillus* and *Bifidobacterium*, and have great potential in improving the safety of fermented products [19,55]. Zou et al. inoculated *B. subtilis* Y4 for solid-state fermentation to improve the quality of Yibin sprouts. The results showed that the inoculation of *B. subtilis* Y4 could promote the growth of *Lactobacillus* and the dominant fungus *Cystofilobasidium*, and reduce the number of *Bacteroides* and *Cryptococcus*. In addition, the inoculation of *B. subtilis* Y4 also increased the content of esters, alkenes, and sweet amino acids in the later stage of fermentation, which helped to improve the quality of Yibin sprouts [56].

#### 2.1.2. Yeast

Yeast is a single-celled fungus that is rich in proteins, carbohydrates, lipids, vitamins, minerals, and a series of enzyme systems and active compounds [2,57]. In the food industry, yeast is mainly used in the field of wine making [58]. Ma et al. found that compared with *Saccharomyces cerevisiae* 85#, *S. cerevisiae* Jiangnan 1# strain enhanced the flavor of rice wine by reducing higher alcohols and increasing acetate. The decrease in higher alcohols is related to the down-regulation of key genes in the Ehrlich pathway, amino acid metabolism, and glycolysis. The increase in acetate content may be attributed to the synergistic interaction between *S. cerevisiae* and other microorganisms and the fermentation environment, which together shape the fermentation kinetics [59,60]. Li et al. used the *Wickerhamomyces anomalus* strain YM001 to biofortify the liquor fermentation process. The results showed that the addition of *W. anomalus* strain YM001 increased the content of total esters in the fermentation samples to a certain extent, especially the content of ethyl acetate, acetic acid, phenylethyl ester, and other compounds, which was of great significance to the improvement of flavor substances [61]. Bian et al. found that the addition of *Saccharomycopsis fibuligera* significantly increased the content of alcohol, organic acid, and amino acid, which gave the yellow rice wine a new characteristic flavor and produced a stronger caramel and vinegar flavor [62]. The fermentation system of a single strain has obvious limitations, and the types and contents of volatile aroma compounds produced are limited. In recent years, researchers have tended to use mixed-strain fermentation strategies (simultaneous fermentation, sequential fermentation) to improve the flavor quality of wine products through the synergistic effect of different yeasts. A number of studies have found that the mixed fermentation of different yeast strains reduces the content of organic acids, produces a wider range of aromatic compounds (especially esters and alcohols), and improves the flavor complexity and sensory quality of fruit wine [63,64,65,66]. Gao et al. found that co-fermentation increased the production of esters and glycerol, but decreased the content of total higher alcohols in the sequential fermentation of wine using *Pichia kluyveri* DG2 and *S. cerevisiae* MT. However, the interaction between different yeast strains is not always synergistically promoted and, in some cases, may have an inhibitory effect [67]. Fu et al. found that in the process of the mixed fermentation of *Candida versatilis* and *Zygosaccharomyces rouxii* to produce soy sauce, *C. versatilis* had a significant inhibitory effect on the growth of *Z. rouxii*, and the inhibitory effect increased with the increase in the initial inoculation amount. This inhibition is likely due to the production of acidic substances by *C. versatilis*, which, in turn, secrete proteases and heat-sensitive substances, inhibiting the growth of *Z. rouxii* [68].

#### 2.1.3. Other Fungi

In addition to yeasts, other fungi such as *Eurotium cristatum* and molds also play an active role in improving food flavor. For example, Song et al. used *Eurotium cristatum* to ferment black tea. The results showed that the bitterness and astringency of black tea were reduced, and the mellowness and floral aroma were significantly enhanced [69]. Yang et al. also used *E. cristatum* to ferment mulberry leaf tea. The results showed that the bitterness and astringency of mulberry leaf tea were also reduced, and the umami and salty taste characteristics were enhanced [70]. In addition, the newly formed compound 1-octen-3-one gives fermented mulberry leaf tea unique mushroom, herbaceous, and earthy flavor characteristics [70]. In terms of legume products, Zhang et al. selected four edible mushroom mycelia to ferment SPI. After the fermentation, the original grass flavor and beany flavor of SPI were reduced; the bitter and astringent characteristics were reduced; the proportion of sweet and tasteless amino acids was increased; and new flavor compounds, benzaldehyde and 5-hydroxymethylfurfural, were formed [71]. Wang et al. also used four kinds of edible fungi to ferment okara in order to reduce the odor volatile compounds. The results showed that the fermentation of edible fungi not only reduced the content of odorous substances such as hexanal and cis-6-nonenal, but also produced linalool, phenylethanol, and other substances, which added new floral, tea, and fruit flavor characteristics to the system [72]. In addition, Yu et al. fermented traditional Japanese smoked and dried meat products using molds. Fermentation improved the color characteristics and taste quality of the product; promoted the production of 3-methyl-1-butanol, 2,5-dimethylpyrazine, and α-pinene; and provided the product with obvious malt, nut, and roasted flavors [73].

Table 1 provides a brief overview of the treatment objects and flavor substances produced through fermentation, and specific flavor regulation directions of several commonly used single-strain fermentations.

#### 2.1.4. Mixed Fermentation

Compared with the simple level of flavor substances in the single-strain fermentation system, mixed-strain fermentation can form a more complex and coordinated flavor substance spectrum through multi-strain synergistic metabolic pathways [75]. Existing studies have confirmed that co-fermentation with different functional composite microbial agents is an effective way to optimize the quality and flavor of fermented food [74,76]. The mixed fermentation technology includes both bacterial and fungal fermentation systems and a mixture of the two.

Bacterial mixed fermentation technology has been widely used in many food processing fields, such as bean products, fermented meat products, pickles, fish products, dairy, and alcoholic beverages. A large number of studies have shown that this fermentation method effectively increased the content of flavor substances (such as diacetyl, 1-heptanol, 1-nonanol) in food, and inhibited the production of undesirable flavor substances (such as hexanal, methyl mercaptan). After the fermentation of bean products using mixed bacteria such as *B. subtilis*, the content of butyric acid and other irritating odorous substances and beany odor-related compounds such as hexanal is reduced [77,78]. In one study, the fermentation of soybean products using *Bacillus bulgaricus* SDL1 and *L. plantarum* Ly8 as starter cultures reduced the content of acetic acid and lactic acid, enhanced the synthesis of branched-chain amino acids and threonine, and increased the content of some esters, acetoin, and pyrazines, thereby significantly improving the flavor characteristics of soybean products [79]. In other research, *L. plantarum* was mixed with *Staphylococcus* and *Bacillus* to ferment sausage and other meat products, which strengthened the hydrolysis process of protein and lipids and promoted the formation of volatile flavor substances such as β-eudesmol, nerolidol, ethyl caproate, and citronellal [80,81,82]. In addition, researchers found that mixed fermentation enhanced the competitive ability of dominant bacteria and inhibited the growth of spoilage bacteria [80,81]. Shan et al. found that *Bacillus* has a general advantage in aroma production over the LAB and *Staphylococcus* used in traditional meat fermentation [82]. In the fermentation of ginger pickles, mixed fermentation rapidly reduced the pH, increased the content of non-volatile organic acids; promoted the formation of volatile compounds, such as olefins, alcohols, phenols, and esters; and gave the ginger pickles a floral, sweet, and sour aroma, thereby improving the overall sensory acceptability [73]. Similarly, in the fermentation of carrots, mixed fermentation not only increased the types and contents of flavor substances such as alcohols, esters, and hydrocarbons, but also reduced the accumulation of nitrite [83]. Peng et al.’s and Hu et al.’s study of the fermentation system of Chinese rice wine and fruit vinegar also confirmed that mixed fermentation can not only improve the acidity of the product, but also leads to a significant increase in volatile components and flavor compounds [84,85]. In terms of fish products, inoculation fermentation effectively inhibited the oxidation of fish fat, resulting in a significant increase in the content of umami amino acids, and some bitter amino acids were inhibited [75,86,87,88,89].

Fungal mixed fermentation is mainly used to improve the flavor characteristics of wine and vegetables. Du et al. used *Monascus purpureus* combined with *L. plantarum* or *S. cerevisiae* to ferment *Porphyra yezoensis* [90]. After fermentation, the protein, total free amino acids, and volatile substances increased significantly, and the concentration of aldehydes decreased. Finally, the mellow, fruity, and sweet aroma of *P. yezoensis* increased, and the fishy and seawater smell decreased [90]. In the fermentation process of yellow rice wine and highland barley wine, the content and type of volatile organic compounds also increased [91,92]. In addition, Peng et al. found that molds increased the content of bitter amino acids during the production of yellow rice wine, but *Saccharomycopsis fibuligera* effectively alleviated these components, thereby enhancing the alcohol body, smoothness, and balance of the beverage [91].

The mixed bacteria and fungi fermentation system is also widely used. In producing protein hydrolysates and soy foods, Cao et al. fermented soybean protein hydrolysates by co-culturing *L. fermentum* and *Pichia fermentum*. The large peptides gradually degraded with fermentation, releasing free amino acids and increasing the umami and sweetness of the protein hydrolysate [93]. Peng et al. fermented soymilk using a mixed culture of LAB and kombucha. The study showed that the mixed starter reduced the content of hexanal, a characteristic flavor substance of soymilk; increased the content of soybean isoflavones and vitamins; and produced new flavor substances, which enhanced the nutritional characteristics of the soymilk and enriched its taste [94]. Qiu et al. used mixed flora (*L. plantarum*, *Pichia guilliermondii*, and *Neurospora crassa*) fermentation to enrich the flavor, reduce the bitterness and beany taste, and improve the freshness and taste of Hongjun tofu [95]. Wu et al. used different halophilic bacteria and yeasts to co-inoculate fermented soy sauce, finding that yeast can promote the growth of bacteria, but bacteria inhibit the growth of yeast. The co-inoculation of *L. fermentum* and *Z. rouxii* showed outstanding performance in flavor enhancement, which strengthened the soy sauce aroma, smoke aroma, and caramel flavor, and improved the umami and bitterness of soy sauce [96,97]. In terms of sauce production, Gao et al. found that the synergistic effect of *L. plantarum*, *Pichia fermentans,* and *Staphylococcus saprophyticus* accelerated the fermentation process of catfish frame fish sauce. The acidity increased, the glucose content decreased, and the umami amino acid content increased, which enhanced the flavor characteristics of the fish sauce [98]. Most studies have found that mixed fermentation using bacteria and fungi (liquid fermentation and solid-state fermentation) increases the type and quantity of flavor substances in alcohol products [99,100,101,102]. Tian et al. found that the complex flora composed of *L. fermentum*, *Rhizopus oryzae*, *S. cerevisiae,* and *W. anomalus* could effectively reduce the content of the harmful substance ethyl carbamate and its precursor urea in Chinese rice wine [100]. Fang et al. and Chen et al. used the mixed fermentation of LAB and yeast to improve the flavor characteristics of sourdough and salt-free fermented wheat gluten [103,104]. The results showed that during the fermentation of sourdough, the accumulation of acetic acid increased significantly, the content of lactic acid and total polyphenols decreased, and the degradation of phytic acid was significantly enhanced [103]. In this fermentation system, the reducing sugar produced by the synergistic metabolism of LAB and yeast can be further utilized by yeast, which may promote the synthesis of flavor substances [103,105]. In addition, Chem et al. further confirmed that there was a significant synergistic effect between LAB and yeast in the biosynthesis of characteristic flavor compounds such as isoamyl acetate [104]. In addition, the mixed fermentation of bacteria and fungi also showed significant effects in improving the flavor of mutton sausage, red yeast rice, hemp seed, and coffee [106,107,108,109,110].

Table 2 presents a brief review of the common treatment objects of three mixed bacteria fermentation approaches, the flavor substances produced through fermentation, and the specific flavor regulation direction.

### 2.2. Possible Regulatory Mechanisms of Microorganisms on Food Flavor

Although significant progress has been made in the study of microbial fermentation to regulate food flavor, there are still obvious deficiencies in current research on the interaction mechanism of strains and the formation and transformation mechanism of flavor substances in food fermentation systems. Understanding the interaction between strains and their metabolic pathways can help optimize the fermentation process parameters and effectively inhibit the reproduction of harmful microorganisms in the fermentation process. After clarifying the formation and transformation mechanism of flavor substances, we can accurately control the synthesis of flavor substances by regulating the culture conditions or genetic modification, and then optimize the whole fermentation process so that the final product presents more coordinated flavor characteristics [111]. These research results can also provide an important theoretical basis for the development of new natural food-flavoring agents.

Liang et al. studied the potential mechanism of the interaction between *L. plantarum* and *Rhodotorula mucilaginosa* in regulating the fermentation process and flavor characteristics of cabbage [112]. The results showed that *L. plantarum*, as a dominant strain, perceives the surrounding environment through quorum sensing signals and upregulates genes related to the synthesis of key compounds, increase in product yield, and formation of biofilm to adapt to the symbiotic environment. On the contrary, *R. mucilaginosa* responded to the stress induced by *L. plantarum* by upregulating the transporters of metabolites, antioxidative-stress-related genes, and longevity regulation, and finally achieved coexistence with *L. plantarum* [112]. In addition, the mixed culture of these two microorganisms significantly increased the production of certain flavor compounds, including lactic acid and acetoin, and reduced bitter amino acids such as phenylalanine, which was attributed to the differentially expressed genes regulated in the proliferation and metabolic pathways of *L. plantarum* [112,113].

Many researchers have conducted in-depth analyses and research on the key metabolic pathways in the process of microbial fermentation. Li et al. used *Schizosaccharomyces pomelo* and *S. cerevisiae* to ferment pomelo wine sequentially. The results showed that the continuous fermentation produced a wider range of aromatic compounds, especially esters and alcohols, which were the key to the aroma of grapefruit wine. Metabolomics analysis further confirmed that amino acid metabolism is the most significant activation pathway, and continuous fermentation is conducive to regulating amino acid levels to minimize the formation of potentially harmful by-products [64]. Gao et al. co-fermented wine using *P. kluyveri* and *S. cerevisiae*, which increased the yield of esters and glycerol and enriched the aroma diversity of wine. Transcriptome analysis showed that 512 genes were differentially expressed in *S. cerevisiae* under co-fermentation conditions, including 318 upregulated genes (mainly related to the synthesis of wine flavor substances) and 194 downregulated genes (mainly involved in glycolysis and amino acid metabolism pathways) [67]. Jia et al. used lactic acid fermentation to improve the flavor of egg white, with the results showing that the fermentation of *Streptococcus thermophilus* increased the content of aldehydes, ketones, alcohols, terpenoids, and aromatic compounds. Metabolic analysis showed that leucine, isoleucine, valine, phenylalanine, glucose, and saturated fatty acids were the key metabolic substrates involved in the formation of aldehydes, alcohols, acids, esters, and ketones during fermentation. Organic acids and their derivatives and organic oxygenates are the most abundant metabolites in egg white. During the 6 h fermentation, the activities of the threonine, methionine, lysine, and isoleucine biosynthetic pathways increased, while the glycolytic pathway was inhibited [114]. Zeng et al. fermented Kuding tea (KDT) using *Aspergillus neoniger* (AN) and *Aspergillus cristatus* (AC), respectively. They found that metabolites such as lipids, phenolic acids, flavonoids, amino acids, and phenolic glycosides were significantly correlated with the flavor characteristics of KDT, and the degradation of phenolic acids and lipids, the deglycosylation of glycoside compounds, and the increase in amino acids were the key to changing the taste characteristics of KDT. In addition, during the fermentation process, AN and AC were involved in five and four metabolic pathways, respectively, and showed different regulatory mechanisms in changing the flavor of KDT. Among them, AN led to the significant deglycosylation of glycosides, while AC affected the degradation of phenolic acids and lipids [115].

In addition, some scholars have also explored the influence mechanism of the microbial fermentation process on flavor substances. Wang et al. studied the mechanism of the effect of non-*Saccharomyces* on the flavor of vinegar. The results showed that the addition of non-*Saccharomyces* during the fermentation process increased the type and content of aromatic esters, produced ethyl acetate and phenethyl acetate, and increased the content of higher alcohols such as isobutanol, phenylethanol, and isoamyl alcohol. The addition of non-Saccharomyces also enhanced the activity of esterase and β-glucosidase. In addition, the concentration of glycerol was increased while ensuring the normal production of alcohol, thus improving the taste and texture of vinegar [116]. Li et al. studied the changes in volatile compounds (VCs) and microbial composition during the co-fermentation of *Lactobacillus sake* with *Micrococcus* and *Lactococcus lactis*, respectively. A total of 123 VCs were identified during the co-fermentation process, of which 25 were core VCs, mainly including aldehydes, alcohols, and ketones. The abundance of most VCs, LAB, and *Lactococcus* increased significantly, while the abundance of *Micrococcus*, *Acinetobacter*, and *Megacoccus* decreased significantly. In addition, *Latilactobacillus* had the greatest influence on the change of core VCs, which played a key role in the improvement of the volatile flavor of fermented surimi [117]. Fan et al. deeply explored the conversion of flavor substances and the synthesis mechanism of B vitamins during the fermentation of chickpea milk using *Lactobacillus*. The results showed that *L. plantarum* FMBL L23251 increased the content of vitamin B3 and vitamin B6 through the L-aspartate acid pathway and 1-deoxy-D-xylulose-5-phosphate-independent pathway. In addition, *L. plantarum* FMBL L23251 effectively removed the beany odor due to its enhanced pyruvate metabolic pathway, and the main aldehydes were converted into the corresponding alcohols or acids while reducing the content of hexanal and 2-pentylfuran [118,119].

**Figure 1:**
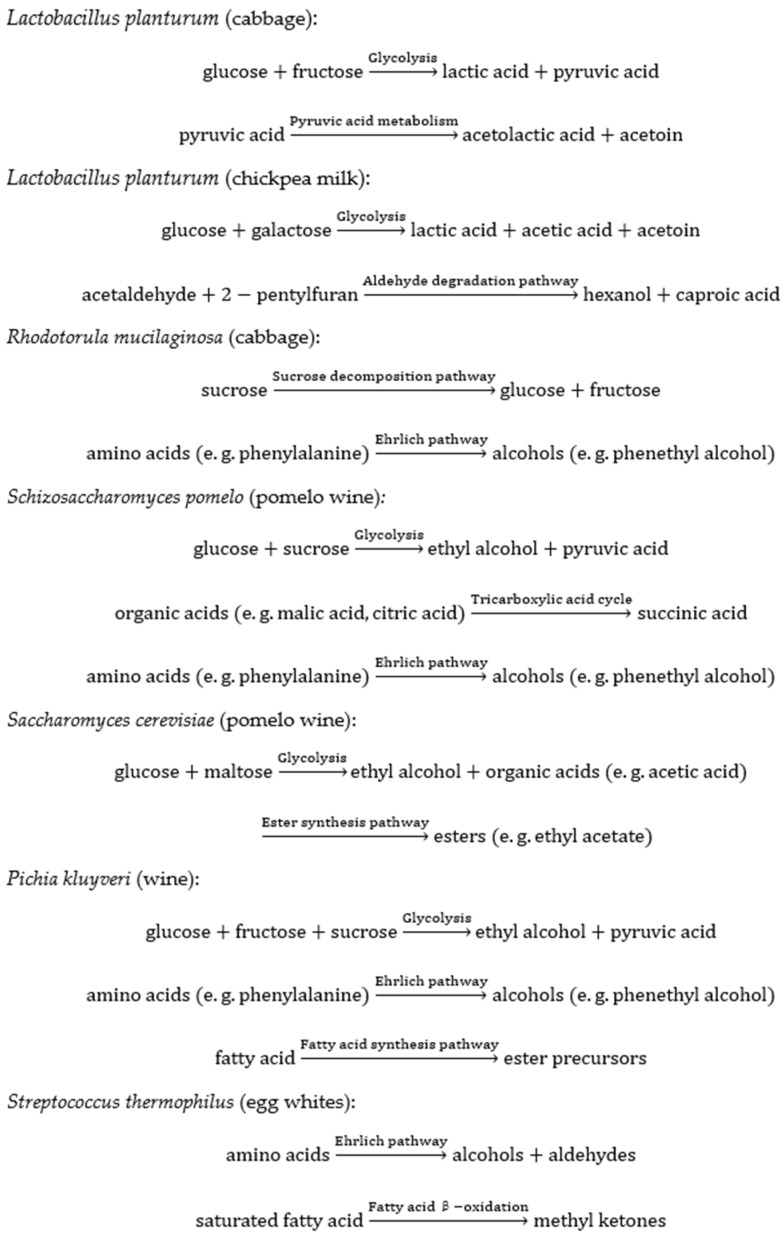
The main substrates, metabolic pathways, and products involved in the regulation of specific food flavor by several major microorganisms.

Figure 1 is a schematic representation of the mechanisms by which some of the major microorganisms regulate the flavor of a particular food.

## 3. Application of Enzyme Catalysis in Regulation of Food Flavor

Microbial fermentation obtains different flavor substances through various metabolic activities of microorganisms. Enzyme catalysis technology has also become another important means to regulate food flavor due to the high efficiency and specificity of enzymes. Since the enzymes found at this stage for regulating food flavor mainly comprise oxidoreductases, transferases, and hydrolases, the following will explain the application of these three types of enzymes in regulating food flavor and their respective catalytic reaction schemes.

### 3.1. Application of Different Enzymes

#### 3.1.1. Oxidoreductases (EC 1)

Oxidoreductases are a class of enzymes that catalyze redox reactions and participate in the synthesis, degradation, or transformation of food flavor substances through electron transfer. Glucose oxidase is a typical oxidoreductase that catalyzes the reaction of β-D-glucose with oxygen to produce gluconic acid and hydrogen peroxide. Existing studies have shown that the enzyme alone or in combination with other enzymes can significantly improve food flavor characteristics [120]. Luo et al. found that the hydrogen peroxide and gluconic acid produced by the glucose oxidase-catalyzed reaction effectively inhibited the release of cooked odorous components in heat-treated melon juice through oxidation and hydrophobicity. In addition, these products reduced the loss of characteristic odor compounds by inhibiting the Maillard reaction, degradation reaction, and oxidation reaction during thermal processing [121]. Liu et al. used the synergistic effect of glucosidase and glucose oxidase not only to inhibit the production of 1-octen-3-one to reduce the cooking taste, but also to increase linalool, benzyl alcohol, phenylethyl alcohol, and nonanal through the metabolism of shikimic acid, glucose, linoleic acid, and linolenic acid, providing the concentrated peach puree with richer grassy and floral flavor characteristics [122]. Botezatu et al. used a complex enzyme system of glucose oxidase and catalase to quickly reduce the pH of high-pH grape juice and unfermented grape juice to the optimal level, while increasing titratable acidity, reducing alcohol content, and preventing oxidation in residual sugar wine [123].

#### 3.1.2. Transferases (EC 2)

Transferase can catalyze the transfer of groups between molecules and affect the aroma and flavor of food by participating in the synthesis, transformation, and modification of flavor substances, which is very important for forming and improving the unique flavor of food. Transglutaminase (TGase) can catalyze the cross-linking reaction between the γ-carboxyamide group of glutamine residue (Gln) and the ε-amino group of lysine residue (Lys) in proteins or peptides to form ε-(γ-glutamyl) lysine isopeptide bonds, thereby changing the secondary structure and function of proteins [124]. The enzyme plays an active role in improving the flavor of fish, beans, and dairy products. Shan et al. found that TGase could induce conformational changes in the polypeptide structure of the Alaska pollock framework protein, which significantly increased the content of umami and sweet amino acids and reduced the content of hydrophobic bitter peptides and hydrophobic amino acids, thus effectively enhancing the umami and reducing the bitterness of the products [125]. During the treatment of hypoallergenic soy protein, the enzyme also showed a significant effect on reducing bitterness [126]. Li et al. combined the application of transglutaminase and pectin fat mimics in the processing of cheddar cheese. The results showed that the treatment not only increased the content of polyunsaturated fatty acids (especially linoleic acid), but also promoted the formation of volatile flavor substances such as alcohols, acids, and methyl ketones [127].

#### 3.1.3. Hydrolases (EC 3)

Hydrolases are a class of enzymes that can catalyze hydrolysis reactions. They are widely present in organisms and participate in physiological processes such as metabolism and digestion [128,129]. The hydrolases commonly used to improve food flavor include proteases, lipases, and amylases [130]. Protease can hydrolyze proteins in food into small molecular peptides and free amino acids, which are the core components of food flavor [131,132]. Researchers have used proteases to improve the flavor of various foods. Studies have found that the addition of enzymes can promote the hydrolysis of meat protein and contribute to the formation of key volatile compounds such as ketones, acids, and esters [133,134]. In the production of cheese [135], oysters and other aquatic products [136], soybean meal and other soybean products [137,138], and plant-based foods [139], the content of free amino acids increased, which effectively improved the umami of food and reduced the beany flavor and other bad flavors of soybean products. The addition of flavor proteases in the production of wheat bread [140] and coconut oil [141] increased the content of soluble sugars and free amino acids, which promoted the Maillard reaction and caramelization reaction and produced more volatile compounds, thereby enriching the flavor of the product. Lipase can hydrolyze fat in food into fatty acids and glycerol [142,143]. Fatty acids can be further oxidized and cleaved to produce aldehydes, ketones, acids, and other flavorful substances to improve food flavor. Chen et al. used the lipase-catalyzed modification of goat milk fat to increase ester content with sweet, floral, and fruity aromas in the final product and decrease the content of FFAs characterized by unpleasant odors, thereby improving the rancidity and tar odor of goat milk [144]. Guan et al. immobilized lipase to promote the release of FFAs in low-fat cheese and improve its flavor [145]. In addition, cellulase, pectinase, amylase, aminopeptidase, and protein glutaminase are also often used to improve the flavor characteristics of products such as fruits, beverages, and protein hydrolysates [146,147,148,149,150,151]. Several studies have confirmed that the recombinant enzyme obtained using a heterologous expression system can also effectively improve the flavor quality of food. Li et al. [152] and Zhao et al. [153] successfully expressed protein glutaminase and lipase in *Escherichia coli*. The former effectively reduced the beany flavor of soy protein, and the latter enhanced the release of fatty acid flavor in low-fat cheese.

The multi-enzyme synergistic catalytic system can effectively degrade the macromolecular substances in the food matrix through multi-level enzymatic reactions and promote the transformation and generation of flavor precursors, thereby significantly improving the diversity and complexity of food flavor substances. A large number of studies have shown that compound enzyme treatment can not only promote the release of various flavor substances in pea textured protein [154], Huangshan floral mushroom protein [155], rapeseed [156,157], and wine [158], but also reduce the unpleasant flavor in boiled pig trotters [159] and low-valued red swamp crayfish (*Procambarus clarkii*) [160], improving the overall edible quality of food.

In recent years, the synergistic application of enzyme catalysis and microbial fermentation technology has become a research hotspot in food flavor regulation. This combined strategy can utilize the high efficiency and specificity of enzyme catalysis to synthesize the key flavor substances required in a short time, and can increase the variety of flavor substances using the diversity of microbial fermentation, making the food flavor richer, more complex, and unique. It was found that this method significantly increased the types and contents of free amino acids, free fatty acids, and flavor substances in fermented foods such as grass carp [161], tartary buckwheat [162], soybean paste [163], and modified cheese [164,165]. Enzyme treatment significantly improved the substrate utilization rate during the processing of sea buckthorn juice. *Schizosaccharomyces pombe* cerevisiae inoculation effectively degraded organic acids and the antioxidant activity of the juice was improved, thereby improving the color quality of the juice [166]. Yan et al. found that enzymatic hydrolysis promotes the production of peptides during the fermentation of soybean meal yogurt, thereby promoting the fermentation of LAB to produce acid, resulting in a shortened fermentation time. Fermentation-assisted enzymatic hydrolysis reduced the bitterness of soybean meal yogurt and also reduced the content of beany flavor components such as 1-pentanol and benzaldehyde [167].

### 3.2. Enzyme Catalytic Reaction Schemes

After understanding the application of three kinds of enzymes in regulating food flavor, the molecular mechanism of enzyme-catalyzed reactions in food processing systems and their regulation of the biosynthetic pathway of characteristic flavor compounds were analyzed. This can not only clarify the essential process of food flavor formation, but also help us to accurately regulate food flavor. The catalytic reaction schemes of oxidoreductases, transferases, and hydrolases are illustrated below.

#### 3.2.1. Possible Catalytic Reaction Schemes by Oxidoreductases (EC 1)

Studies have found that lipoxygenase helps to promote the production of flavor substances in food. Ye et al. found that lipoxygenase can specifically catalyze the oxidation of polyunsaturated fatty acids with a cis, cis-1,4-pentadiene structure to form hydroperoxides with conjugated double bonds. The hydroperoxide is unstable and will continue to oxidize and decompose, producing volatile flavor substances such as hydrocarbons, alcohols, and aldehydes, such as 1-octene-3-ol and hexanal. These substances give fermented fish products a unique flavor [20]. Wang et al. also found a similar conclusion when treating flounder bone oil with soybean lipoxygenase. They found that soybean lipoxygenase can catalyze the oxidation of polyunsaturated fatty acids at the ω-6 carbon position to produce specific products such as 15-hydrogen peroxide-EPA and 17-hydrogen peroxide-DHA, which are then decomposed into volatile compounds (such as 1-penten-3-one, (E)-2-hexenal and 2-pentylfuran), thereby improving the flavor of the product [168]. The reaction scheme of lipoxygenase is shown in Scheme (1):

(1) $${\;C}_{18}H_{32}O_{2}+{\;O}_{2}\overset{\mathrm{lipoxygenase}}{\to }CH_{3}\text{-}{(CH_{2})}_{4}\text{-}\mathrm{CH}=\mathrm{CH}\text{-}CH_{2}\text{-}\mathrm{CH}=\mathrm{CH}\text{-}{(CH_{2})}_{7}\text{-}\mathrm{COOH}\to CH_{2}=\mathrm{CH}\text{-}\mathrm{CH}\left(\mathrm{OH}\right)\text{-}{(CH_{2})}_{4}\text{-}CH_{3}+CH_{2}=\mathrm{CH}\text{-}{\left(CH_{2}\right)}_{7}\text{-}\mathrm{COOH}\;$$

Recent studies have shown that laccase can effectively remove the bad flavor substances in food. Spaccasassi et al. found that laccase could effectively reduce the content of the bitter compound kaempferol 3-O-(2-O-sinapoyl-β-D-sophoroside) in rapeseed protein. In addition, laccase can also catalyze the oxidative polymerization of polyphenols such as sinapic acid, change the structure and properties of polyphenols, reduce bitter and astringent substances, and thus improve the flavor of rapeseed protein [169]. The reaction scheme of laccase is shown in Scheme (2):

(2) $$R\text{-}C_{6}H_{4}\left(\mathrm{OH}\right)+\frac{1}{2}O_{2}\overset{\mathrm{laccase}}{\to }R\text{-}C_{6}H_{4}O+H_{2}O$$

#### 3.2.2. Possible Catalytic Reaction Schemes by Transferases (EC 2)

Studies have shown that transferases play an active role in improving the flavor of protein hydrolysates. Su et al. used TGase to significantly increase the umami intensity of pea protein isolate hydrolysate (PPIH) by 50% and reduce the bitterness intensity by 40%. They found that after TGase treatment, the proportion of umami amino acids (glutamic acid, aspartic acid), hydrophilic amino acids (threonine, serine, cysteine, glycine, histidine, lysine), and proline in non-free amino acids increased significantly, while the proportion of hydrophobic amino acids (alanine, valine, leucine, isoleucine, methionine, and phenylalanine) decreased significantly. During the TGase catalytic process, more umami/hydrophilic amino acids and proline may be involved in the formation of peptides, and the newly formed peptides have a higher binding affinity with the umami receptors T1R1-T1R3 in PPIH than the original peptides. In addition, compared with the short peptides in the untreated PPIH, TGase treatment promoted the production of long peptides, which were enriched in umami and hydrophilic amino acids, resulting in enhanced umami and reduced bitterness of the hydrolysate [170]. Shan et al. also found similar results when using TGase to improve the flavor of Alaska pollock steak hydrolysate. The increase in umami and sweet amino acids, the decrease in hydrophobic amino acids, and the change in the composition ratio in the peptide group increase the umami while reducing the bitterness, thereby improving the taste characteristics of the product and making it more palatable as a whole [125]. The reaction scheme of TGase is shown in Scheme (3):

(3) $$R_{1}\text{-}\mathrm{CONH}\text{-}\mathrm{CH}R_{2}\text{-}\mathrm{CO}\text{-}R_{3}+H_{2}O\overset{\mathrm{transglutaminase}}{\to }R_{1}\text{-}\mathrm{CO}\text{-}\mathrm{CH}R_{2}\text{-}\mathrm{CO}\text{-}R_{3}+NH_{3}$$

Xia et al. enhanced the flavor of *Pleurotus geesteranus* protein hydrolysate by using *Bacillus amyloliquefaciens* γ-glutamyl transpeptidase. They found that γ-glutamyl transpeptidase can catalyze the reaction of glutamine (Gln) and receptors (free amino acids or peptides) to produce gamma-glutamyl peptides. γ-glutamyl peptide can induce kokumi taste through calcium-sensing receptor (CaSR) recognition [171]. In addition, compared with the blank chicken soup model, the umami chicken soup model and the salty chicken soup model, the umami and salty taste of the chicken soup model with the γ-glutamyl hydrolyzate prepared by *P. geesteranus* increased significantly, which indicated that the γ-glutamyl peptide played a synergistic role with the original flavor substances of chicken soup and improved the flavor [171]. The reaction scheme of γ-glutamyl transpeptidase is shown in Scheme (4):

(4) $$H_{2}N\text{-}\mathrm{CH}\left(\mathrm{COOH}\right)\text{-}{\left(CH_{2}\right)}_{2}\text{-}\mathrm{CON}H_{2}+H_{2}N\text{-}CH_{2}\text{-}\mathrm{COOH}\overset{\gamma -\mathrm{glutamyl}\;\mathrm{transpeptidase}}{\to }H_{2}N\text{-}\mathrm{CH}\left(\mathrm{COOH}\right)\text{-}{\left(CH_{2}\right)}_{2}\text{-}\mathrm{CO}\text{-}\mathrm{NH}\text{-}CH_{2}\text{-}\mathrm{COOH}+NH_{3}\;$$

#### 3.2.3. Possible Catalytic Reaction Schemes by Hydrolases (EC 3)

In a study of the flavor regulation of fish products, Wang et al. showed that the addition of lipase could significantly promote the formation of key flavor substances such as 1-octene-3-ol, hexanal, and nonanal in lightly salted large yellow croaker. The reaction scheme is that lipase can effectively catalyze the hydrolysis reaction of triacylglycerol and phosphatidylethanolamine, resulting in a large number of flavor precursors, including intermediate products such as lysophosphatidylcholine and diglyceride [172]. Ye et al. used lipase and lipoxygenase to treat fermented fish products and found that lipase can catalyze the hydrolysis of lipids to produce free fatty acids. These free fatty acids are oxidized by lipoxygenase to form alcohols (such as 3-methyl-1-butanol) and aldehydes (such as valeraldehyde, hexanal, heptanal, and octanal), thereby giving more flavor to fish products [20]. The reaction scheme of lipase is shown in Schemes (5) and (6):

(5) $${\;C}_{57}H_{104}0_{6}+{3H}_{2}O\overset{\mathrm{lipase}}{\to }{3C}_{18}H_{34}O_{2}+C_{3}H_{8}O_{3\;}$$

(6) $$R_{1}\mathrm{COOC}H_{2}\mathrm{CH}(\mathrm{OOC}R_{2})CH_{2}\mathrm{OPO}(\mathrm{OH})\mathrm{OC}H_{2}CH_{2}NH_{2}+2H_{2}O\overset{\mathrm{lipase}}{\to }\mathrm{HOC}H_{2}\mathrm{CH}(\mathrm{OH})CH_{2}\mathrm{OPO}(\mathrm{OH})\mathrm{OC}H_{2}CH_{2}NH_{2}+R_{1}\mathrm{COOH}+R_{2}\mathrm{COOH}$$

Yang et al. found that phospholipase promoted the oxidative degradation of phospholipids by regulating the glycerophospholipid metabolic pathway, thereby increasing the content of volatile flavor substances such as aldehydes and ketones and reducing the accumulation of unpleasant flavor substances, thereby improving the overall flavor of fish [173]. The reaction scheme of phospholipase is shown in Scheme (7):

(7) $$\begin{array}{c} R_{1}\mathrm{COOC}H_{2}\mathrm{CH}\left(\mathrm{OOC}R_{2}\right)CH_{2}\mathrm{OPO}\left(\mathrm{OH}\right)\mathrm{OC}H_{2}CH_{2}N{\left(CH_{3}\right)}_{3}+2H_{2}O\overset{\mathrm{phospholipase}}{\to }\mathrm{HOC}H_{2}\\ \;\;\;\;\;\;\;\;\;\;\;\;\;\;\;\;\;\;\;\;\;\;\;\;\;\;\;\;\;\;\;\;\;\;\;\;\;\;\;\;\;\;\;\;\;\;\;\;\;\;\;\;\;\mathrm{CH}\;\left(\mathrm{OH}\right)CH_{2}\mathrm{OPO}\left(\mathrm{OH}\right)\mathrm{OC}H_{2}CH_{2}N{\left(CH_{3}\right)}_{3}+R_{1}\mathrm{COOH}+R_{2}\mathrm{COOH} \end{array}$$

In the field of protein food flavor regulation, Liao et al. used complex enzymes to produce more of the flavor substance (E, E)-2,4-decadienal, which greatly improved the overall aroma of chicken soup [174]. Fan et al. studied the compensation mechanism of the synergistic effect of a cell-free extract and enzyme system on cheese flavor. The results showed that the combined treatment of neutral protease and flavor protease increased the content of total leucine, valine, and isoleucine, and the soluble nitrogen content reached the level of traditional 12-month mature cheese. Moreover, due to the flavor compensation effect of transaminase and decarboxylase converting leucine to 3-methylbutanal, and keto acid dehydrogenase and aldehyde dehydrogenase converting 3-methylbutyrate to 3-methylbutanal, the content of volatile flavor substances in enzyme-modified cheese is close to the level of cheese matured for 12 months [175]. Chen et al. used enzymatic hydrolysis and fermentation to treat egg white protein. The results showed that enzymatic hydrolysis and fermentation promoted the production of lipids and umami amino acids, respectively, and both can promote protein aggregation. In addition, enzymatic hydrolysis destroyed the hydrophobic cavity bound to the off-flavor compounds, released the off-flavor compounds bound to the protein, and then removed them through fermentation to achieve the deodorization of egg white powder [176]. The reaction scheme of proteinase is shown in Scheme (8):

(8) $${\;H}_{2}N\text{-}{\left(\mathrm{CHR}\text{-}\mathrm{CO}\text{-}\mathrm{NH}\right)}_{n}\text{-}\mathrm{CHR}\text{-}\mathrm{COOH}+nH_{2}O\overset{\mathrm{proteinase}}{\to }nH_{2}N\text{-}\mathrm{CHR}\text{-}\mathrm{COOH}\;\;\;\;$$

**Figure 2:**
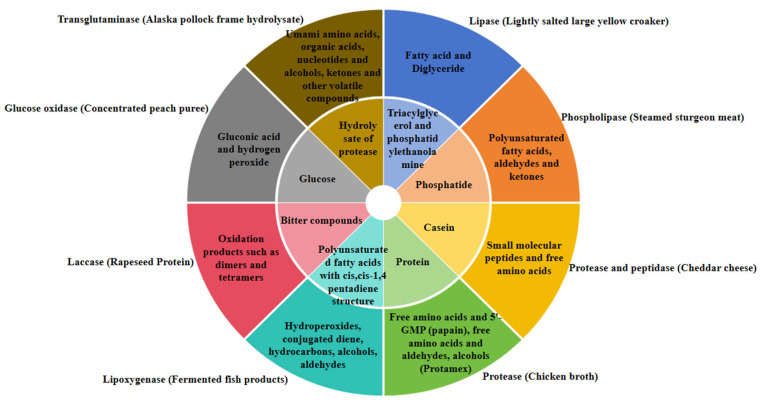
Several commonly used oxidoreductases, transferases, and hydrolases catalyze substrates and corresponding products when regulating the flavor of specific products. The inner ring is the substrate and the outer ring is the product.

Figure 2 shows the substrates and products of several enzymes commonly used in improving food flavor.

## 4. Conclusions

In this study, the application and mechanism of microbial fermentation and enzyme catalysis in food flavor regulation were systematically described. Different microorganisms play a unique role in microbial fermentation. Bacteria such as LAB and *Bacillus* can promote the decomposition of proteins and lipids, produce flavor precursors, enhance food flavor, and enhance safety. Yeast is widely used in the field of brewing [177]. *Aspergillus*, mold, and other fungi can reduce unpleasant flavors in food and enhance new flavor characteristics. Mixed-strain fermentation can form a more complex and coordinated flavor profile through multi-strain synergistic metabolic pathways. The mechanism of microbial fermentation to regulate flavor mainly involves the interaction between strains and the formation and transformation of flavor substances. Different microorganisms interact with each other through quorum sensing and gene regulation, participate in metabolic pathways such as amino acids and glycolysis, and produce diverse flavor substances (such as esters, aldehydes, and ketones). Enzyme catalysis is also indispensable in the regulation of food flavor. Oxidoreductase affects food flavor by catalyzing redox reactions. Transferases are involved in the synthesis and transformation of flavor substances. For example, transglutaminase can change protein structures, enhance umami, and reduce bitterness. Hydrolases can hydrolyze the macromolecular substances in food into small molecular flavor components. The reaction scheme of enzyme catalysis is to promote the formation and transformation of flavor precursors by catalyzing specific chemical reactions, thereby improving food flavor. For oxidoreductases, lipoxygenase can catalyze the oxidation of polyunsaturated fatty acids to produce hydroperoxides, which are further oxidized and decomposed to form volatile flavor substances such as hydrocarbons and alcohols. Laccase can catalyze the oxidative polymerization of polyphenols such as sinapic acid in rapeseed protein, thereby reducing the amount of bitter and astringent substances. For transferases, transglutaminase can improve the flavor of food by catalyzing intramolecular or intermolecular cross-linking of proteins and reactions between proteins and amino acids (such as glutamic acid). γ-glutamyl transpeptidase can catalyze the reaction of glutamine with free amino acids or peptides to produce γ-glutamyl peptides. For hydrolases, lipases can catalyze the hydrolysis of lipids to produce free amino acids. Phospholipase can promote the hydrolysis of phospholipids to produce fatty acids, which can be further oxidized to aldehydes. Protease can hydrolyze proteins into peptides and amino acids, and some of the amino acids produced are umami and sweet, which can enhance the flavor of food.

Microbial fermentation and enzyme catalysis technology have shown great potential in the field of food flavor regulation due to their environmentally friendly and efficient characteristics. Future research may focus on the following suggested points to widen the application of related technologies in the food industry and meet consumer demand for high-quality food flavor.

(1) Microorganisms are essential for the improvement of food flavor through fermentation. Therefore, the isolation and identification of flavor-producing microbial strains from traditional fermented food can be a potential way to enrich the microbes used in food. The construction of engineered strains for the recombinant expression of flavor-producing enzymes in food-safe microorganisms is also an alternative method.
(2) Since the compounds in fermented food are complex, the identification of those compounds essential for food flavor is beneficial for quality control during food fermentation. The relationship between fermentation conditions and food flavor should also be explored in order to optimize standard protocol in food factories.
(3) The enzymatic transformation of food to improve flavor is highly efficient. However, some flavor compounds are still extracted from plants or chemically synthesized. It is necessary to explore or design novel enzymes for the production of new natural flavor agents or low-cost and environmentally friendly synthesized flavors.
(4) For the industrial application of enzymes in food flavor regulation, it is important to investigate food-safe, low-cost immobilization supports with good catalytic performance. The design of enzymatic reactors for food flavor improvement should also be explored.
(5) Since an increasing number of flavor compounds produced by microorganisms and enzymes have been identified and their functions revealed, a database of flavor compounds, functions, producers, and transformers should be established; moreover, new flavor compounds could be designed with the assistance of AI, providing sufficient learning and training has been conducted. Further, potential pathways for the synthesis of new flavor compounds using microbial and/or enzymatic methods may also be identified by AI models based on the database.

## Figures and Tables

**Table 1:** The treatment objects of the fermentation of different strains, the flavor substances produced through fermentation, and the specific direction of flavor regulation.

Microbial Type	Name	Processing Object	Flavor Substances	Odor	Reference
Bacteria	*L. planturum*	Asparagus juice	1-Octen-3-ol, b-damascenone, 1-Octen-3-one, and dimethyl trisulfide	Enhanced mushroom and floral flavor Reduced green flavor	[74]
	*L. planturum*	Surimi product	Ethyl octanoate	Produced a sweet and pleasant brandy aroma	[36]
	*L. planturum*	Dried-fermented radish	Lactic acid, oxalic acid, glutamic acid (Glu), ocimenol, and trans-4-thujanol	Produced fruit, floral, and grassy flavors	[38]
	*L. planturum*	Shiitake mushrooms	5′-nucleotides, cysteine, histidine, citric acid, and tartaric acid	Enhanced umami	[42]
	*B. subtilis*	Yibin Yacai	β-Myrcene, Germacrene D, γ-Elemene, ethyl Cinnamate, ethyl p-methoxycinnamate, and sweet amino acids	Enhanced fruit, herb, green, oil, wood, and aroma flavors	[57]
Fungi	*S. cerevisiae*	Huangjiu	Acetate esters	Enhanced aroma	[60]
	*W. anomalus*	Baijiu	Ethyl acetate	Enhanced the aftertaste, reduced dryness and astringency	[62]
	*E. cristatum*	Black tea	β-damascenone, 2-ethyl-5-methylpyrazine, acetophenone, and tetrahydro-α, α, 5-trimethyl-5-vinylfuran-2-methanol	Enhanced floral, honey, sweet flavor Reduced bitterness and astringency	[70]
	Mold	Smoked-dried bonito	3-Methyl-1-butanol, 2,5-dimethylpyrazine, and α-pinene	Produced malt, nutty, roasted aromas	[74]

**Table 2:** The common treatment objectives of three mixed bacteria fermentation approaches, the flavor substances produced through fermentation, and the specific flavor regulation direction.

Category	Microorganism	Processing Object	Flavor Substances	Odor	Reference
Bacterial mixed fermentation	*B. velezensis* and *L. plantarum*	Fermented soybean foods	Acetoin and pyrazines	Enhanced flavors	[73]
	*L. plantarum* and *Staphylococcus warneri*	Fermented meat rice	Ethyl hexanoate, β-eudesmol, nerolidol, ethyl caproate, and citronellal	Enhanced flavors	[80]
	*Leuconostoc mesenteroides*, *Weissella cibaria*, and *L. plantarum*	Ginger pickle	Eucalyptol, α-terpinene, 1-hexanol, 2,4-di-tert-butylphenol, methyl geranate, and (−)-lavender acetate	Produced floral, sweet, and sour fragrance	[74]
	*Tetragenococcus muriaticus*, *B. subtilis*, and *Staphylococcus edaphicus*	Low-salt fish sauce	1-heptanol, 1-nonanol, 1-octanol, ethyl acetate, and 2-pentylfuran	Enhanced floral, mushroom, fruity, and grassy flavors Reduced pungent, sulfur, and fishy flavors	[75]
Fungal mixed fermentation	*M. purpureus* and *S. cerevisiae*	*Pyropia yezoensis*	Glu, ethanol, and ethyl acetate	Enhanced alcohol, fruity, and sweet aromas Reduced fishy, grass, and seawater odors	[90]
	*Rhodotorula mucilaginosa* and *S.cerevisiae*	Cider	Isoamyl acetate and phenethyl acetate	Enhanced fruity and floral notes	[66]
	*Candida glabrata* and *S. cerevisiae*	Blueberry wine	Isoamylol, 2-phenylethanol, isoamyl acetate, ethyl acetate, and ethyl laurate	Enhanced sweetness, fruitiness, and floral flavors	[63]
Mixed bacteria and fungi fermentation	*L. fermentum* and *P. fermentans*	Soybean protein hydrolysates	Umami amino acids, lactic acid, acetic acid, and malic acid	Enhanced saltiness, umami, and sweetness Reduced bitterness	[93]
	*N. crassa*, *L. plantarum* and *Meyerozyma guilliermondii*	HongJun tofu	Glu, aspartic acid, γ-aminobutyric acid, and 5′-nucleotides	Enhanced fruity, mushroom, floral, and nutty flavors Reduced bitterness	[95]
	*Pichia anomala* and *L. plantarum*	Chi-flavor baijiu	Phenylethyl alcohol, (E)-2-octenal, and diethyl succinate	Enhanced flavors	[99]
	*S. cerevisiae* and *B. licheniformis*	Jujube wine	Isoamyl acetate and phenethyl acetate	Enhanced flavors	[101]

## Data Availability

No new data were created or analyzed in this study. Data sharing is not applicable to this article.
